# Electrochemical Sensing of Urinary Chloride Ion Concentration for Near Real-Time Monitoring

**DOI:** 10.3390/bios13030331

**Published:** 2023-02-28

**Authors:** Anna M. Nelson, Sanaz Habibi, John O. L. DeLancey, James A. Ashton-Miller, Mark A. Burns

**Affiliations:** 1 Department of Chemical Engineering, University of Michigan, Ann Arbor, MI 48109, USA; 2 Department of Obstetrics & Gynecology, University of Michigan, Ann Arbor, MI 48109,USA; 3 Department of Mechanical Engineering, University of Michigan, Ann Arbor, MI 48109, USA; 4 Department of Biomedical Engineering, University of Michigan, Ann Arbor, MI 48109, USA

**Keywords:** urinary chloride detection, electrochemical sensor, chronopotentiometry

## Abstract

Urinary chloride concentration is a valuable health metric that can aid in the early detection of serious conditions, such as acid base disorders, acute heart failure, and incidences of acute renal failure in the intensive care unit. Physiologically, urinary chloride levels frequently change and are difficult to measure, involving time-consuming and inconvenient lab testing. Thus, near real-time simple sensors are needed to quickly provide actionable data to inform diagnostic and treatment decisions that affect health outcomes. Here, we introduce a chronopotentiometric sensor that utilizes commercially available screen-printed electrodes to accurately quantify clinically relevant chloride concentrations (5–250 mM) in seconds, with no added reagents or electrode surface modification. Initially, the sensor’s performance was optimized through the proper selection of current density at a specific chloride concentration, using electrical response data in conjunction with scanning electron microscopy. We developed a unique swept current density algorithm to resolve the entire clinically relevant chloride concentration range, and the chloride sensors can be reliably reused for chloride concentrations less than 50 mM. Lastly, we explored the impact of pH, temperature, conductivity, and additional ions (i.e., artificial urine) on the sensor signal, in order to determine sensor feasibility in complex biological samples. This study provides a path for further development of a portable, near real-time sensor for the quantification of urinary chloride.

## 1. Introduction

Urinary chloride is a versatile, readily available biomarker for diagnosing and monitoring a wide variety of diseases involving kidney and circulatory health. As the most abundant extracellular anion [1], chloride accounts for 70% of the total anion concentration in the body [2,3], and is responsible for regulating the extra/intracellular volume [1], maintaining acid/base equilibrium [1,2,4,5], and upholding muscular activity [2,4]. As a mobile ion that is primarily regulated through kidney excretion [6,7], urinary chloride is physiologically dynamic. Timed spot and 24-h sample collection are often needed to account for intra- and interday variations and to capture ion changes [8], which can aid in early disease detection and monitor organ health. Specifically, chloride concentration has been shown to be an indicator for prerenal acute kidney disease [1], acid-base disorders [9,10], dietary salt intake [10,11,12,13,14], and acute heart failure [5,15]. Recently, lower chloride concentrations (<53 mM) have been linked to higher mortality in critically ill patients in the intensive care unit (ICU) [4]. To quantify and monitor chloride ion concentration, a chloride sensor is needed to quickly detect ion change. Thus, a near real-time sensor could accelerate detection of disease, monitor organ health, and inform treatment decisions.

Traditional analytical techniques have been employed as the gold standard to accurately quantify chloride concentration. Analytical techniques include ion chromatography [16,17], colorimetry [7,13,14], fluorescence [16], and potentiometric ion selective electrodes (ISE) [18,19,20]. Potentiometric ISEs are most commonly employed in electrolyte analyzers used in hospital settings [11], as they are accurate and reliable; however, they rely on a consistent reference electrode, which is difficult to miniaturize, requires an internal solution, and requires constant recalibration to remain stable [18]. Electrolyte analyzers are capable of accurately quantifying multiple ions, but tend to be expensive, have a large footprint, and require time-consuming calibration. Furthermore, analyzers require processing samples in a certified lab, which can be a time-consuming process. Thus, simple and cost-effective sensors are needed to achieve near real-time chloride monitoring. For example, such sensors could be integrated into a wearable uroflowmeter [21].

Optical chemosensors [7,13,22,23] and electrochemical sensors [24,25,26,27,28,29,30] have been investigated for cost effective, near real-time chloride detection. Optical chemosensors are typically paper-based sensors [14,16,31] (e.g., dipsticks); due to their portability, they have been widely used in qualitative field studies [18]. However, optical chemosensors are typically single use, and often require adding reagents (such as fluorescent probes, quenching agents) to obtain results. Thus, they are not suitable for long-term near real-time monitoring of urinary chloride.

Electrochemical sensors are often reusable, and can be used for long term measurement [18]. Aside from potentiometric ISEs, common electrochemical techniques include electrochemical impedance spectroscopy (EIS) [30,32,33], voltammetry [24,25,34,35], and chronopotentiometry [26,27,28]. EIS is an emerging technique used in development of sweat chloride sensors [29,36,37,38] where complex modification of the electrode surface (e.g., ionophores) is required to achieve chloride selectivity. Voltammetric sensors also may need complex chemical surface modification, and often detect a smaller resolvable range; however, they can detect low concentrations of chloride (10^−6^ M) [18]. Chronopotentiometric sensors are advantageous, as they rely on measuring a potential shift and not an absolute potential; thus, a pseudo-reference electrode can be used. The pseudo-reference electrode only needs to maintain stability during short measurement times, which makes the sensor ideal for miniaturization and long-term measurement [28]. To the authors’ knowledge, chronopotentiometric sensors have never been optimized for detecting urinary chloride, but the technique shows promise for developing a portable and near real-time urinary chloride sensor, with no required sample preparation.

In this study, we developed a chronopotentiometric sensor capable of measuring a physiologically relevant range [4,8] of chloride ion concentrations (5–250 mM), using simple silver electrodes with no surface modification or added reagents. We studied the influence of the chloride ion concentrations effect on the sensor signal, and identified current densities that can resolve different concentration ranges. To determine the optimal current density for a particular chloride concentration, we electrically and optically characterized the surface of the working electrode by interpreting chronopotentiograms, scanning electron microscopy (SEM) images, and energy dispersive spectroscopy (EDS) spectra. We developed a novel current density sweeping algorithm that allows resolution of the relevant chloride concentration range with a single sensor in 3 s (25–200 mM). Furthermore, we demonstrated reusable sensors that could be suitable for long-term measurement. Lastly, we investigated the effect of pH (4.4–7.7), temperature (25–37 °C), conductivity (1.1–7 S/m), and additional ions (i.e., artificial urine) on the chronopotentiometric signal of our sensor.

## 2. Theory

**Figure 1:**
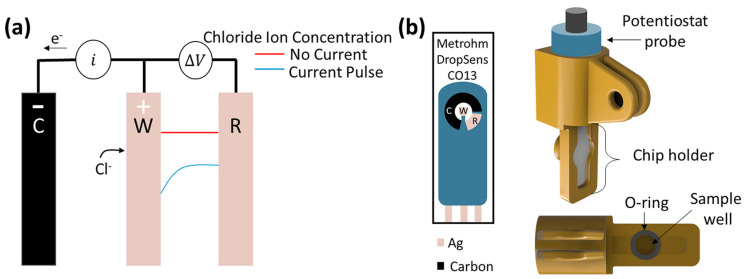
(**a**) Schematic of chronopotentiometric method. The local depletion creates a chloride ion concentration gradient, shown by the blue curve, between the working and reference electrodes. (**b**) Schematic of DropSens C013 screen-printed electrodes and chip holder. The working electrode (W) and reference electrode (R) are silver, while the counter electrode (C) is made of carbon.

As shown in Figure 1a, a constant current is applied between the working and counter electrode of the sensor, and the applied current induces a faradaic reaction on the surface of the working electrode. The silver working electrode will react with chloride ions in the electrolyte to form a solid silver chloride deposit, as shown in Equation (1):

Ag(s) + Cl^−^(aq) → AgCl(s) + e^−^ (1)

When the mass transport of the chloride ions from the bulk solution to the working electrode surface is slower than the faradaic reaction, the chloride ions will start to locally deplete [39]. The local depletion creates a chloride ion concentration gradient between the working and reference electrodes, represented by the blue curve in Figure 1a. The concentration gradient is derived from the migrative and diffusive terms of the Nernst–Planck equation [26,39]. The derivation of the concentration gradient assumes the convective terms are negligible, as the system is in stagnant operating conditions. When the local chloride concentration is completely depleted at the surface of the working electrode, the ion flux to the surface will no longer be upheld, and the potential will rapidly increase to find a new faradaic process to fulfill the current [27,40]. In chronopotentiometry, the potential response versus time (chronopotentiogram) is recorded while the current is applied, and the complete local chloride depletion at the surface is indicated as an inflection point in the chronopotentiogram [27]. The inflection point can be quickly identified by finding the maximum of the derivative of the chronopotentiogram. The corresponding time at the inflection point is known as the transition time (τ), and is defined by the Sand equation (Equation (2)) [27,40]:

(2) $$\tau =D\pi \;{\left[\frac{{\mathrm{FC}}^{*}}{2j\left(1-t_{{\mathrm{Cl}}^{-}}\right)}\right]}^{2},$$

(3) $$t_{{\mathrm{Cl}}^{-}}=\frac{|z_{Cl^{-}}|\lambda_{Cl^{-}}C_{Cl^{-}}^{*}}{\sum_{k}|z_{k}|\lambda_{k}C_{k}^{*}}$$

where F is Faraday’s constant, D is the mean chloride ion diffusion coefficient, j  is the current density, $C^{*}\;$ is the bulk chloride concentration, and $t_{{\mathrm{Cl}}^{-}}$ is the transference number of chloride, or the fraction of chloride ions per total ions in solution. The chloride ion transference number can be calculated (Equation (3)) for chloride (${\mathrm{Cl}}^{-}$) and other ion species (k) present in the electrolyte, where z is the charge of the ion,  λ is the limiting molar conductivity, and $C^{*}$ is the bulk ion concentration. To calculate the transference number and diffusion coefficient, values for limited ion conductivities [41] at 25 °C and diffusion coefficients [42] at infinite dilution in water at 25 °C were used. In the presence of many ions that are not chloride (e.g., high conductivity of supporting electrolyte), $\;t_{{\mathrm{Cl}}^{-}}$ is negligible. The bulk chloride ion concentration directly relates to the transition time, and can be used as the chloride sensor signal [27].

Resolution of the transition time at different chloride concentrations can be difficult to obtain at improper current densities. The current density directly relates to the rate of the reaction, and can influence how fast the silver chloride reaction is occurring. If a chloride concentration is too large for a particular current density, the system cannot fully deplete the local chloride ions because the reaction rate is too slow. Alternatively, if a chloride concentration is too small for a particular current density, the reaction and transition time will occur too quickly. When the transition time occurs at time scales that are too short (τ < 10 ms), a significant portion of the applied current goes towards charging the double layer, and thus inaccurately captures chloride depletion [27,40]. At longer time scales (τ > 6 s), convective effects from ambient vibrations, or natural convection resulting from density gradients at the electrode surface, can no longer be ignored, and can influence the chloride ion concentration gradient [27,40]. Thus, a chosen current density needs to resolve a variety of chloride concentrations within approximately 0.1 to 6 s, in order to accurately reflect the Sand equation (Equation (2)).

## 3. Materials and Methods

### 3.1. Chemicals and Test Solution Preparation

Sodium sulfate (Na_2_SO_4_, >99%), sodium chloride (NaCl, >98%), and citric acid (C₆H₈O₇, >99.5%) were purchased from Sigma-Aldrich (St. Louis, MO, USA). Sodium hydroxide (5 N NaOH) was purchased from EMD Millipore (Burlington, MA, USA). Customized artificial urine without chloride was purchased from Biochemazone (BZ104, Edmonton, AB, CA) at a pH of 6.3. The purchased artificial urine contained sodium citrate, sodium phosphate dibasic, magnesium sulphate hepta hydrate, sodium sulfate, potassium phosphate, uric acid, urea, acetic acid, and creatinine.

The simple solutions were made by creating a stock solution of 20 mM Na_2_SO_4_ with deionized water, and adding NaCl to adjust the chloride to a range of 5 to 250 mM, the relevant range present in urine. The simple solutions had a pH of 5.9, and conductivity varied with the NaCl concentration in a relevant range of 0.4 to 3.5 S/m [43]. For pH experiments, the pH of simple solutions was adjusted with a 5% citric acid solution and 5 N sodium hydroxide solution, and sat overnight until the solutions were 4.7 and 7.7. The pH was measured with a pH meter (Thermofisher, Waltham, MA, USA). For conductivity experiments, the conductivity was adjusted by changing the Na_2_SO_4_ concentration (5–500 mM). The conductivity of the solutions was measured with a conductivity meter (Thermofisher, Waltham, MA, USA). For artificial urine samples, artificial urine was spiked with NaCl to simulate relevant chloride concentrations (50–150 mM).

### 3.2. Sensors and Experimental Setup

Chronopotentiometry was used to detect the chloride concentration in simple and artificial urine samples. To this end, screen printed electrodes, shown in Figure 1b, (DS-C013, Metrohm Dropsens, Oviedo, Spain) containing a silver working electrode, a carbon counter electrode, and a silver pseudo-reference electrode were used to perform all experiments. The working electrode had a 1.6 mm diameter, with an area of 2 mm^2^.

The sensors were inserted into a custom 3D-printed chip holder with a 300 µL fluid well. The chip holder, shown in Figure 1a, was fabricated from biocompatiable dental SG resin (Formlabs, Sommerville, MA, USA) and treated with a ceramic hydrophobic chemical (NS 605, Nanoslic, Cincinnati, OH, USA) for better sealing. Solutions were pipetted into the fluid well, and an O-ring was placed in the holder to prevent solution leakage. The screen-printed electrodes were inserted into the chip holder and contacted the solution. The solutions were not stirred, and were preheated in the water bath prior to testing. The chip holder was submerged in a water bath to control temperature of the solutions during chronopotentiometry experiments. The electrodes were connected to a SP- 200 potentiostat (Biologic Science Instruments, Seyssinet-Pariset, France). The working, counter, and reference electrodes were connected to the corresponding potentiostat terminals. The circuitry was manipulated using Biologic’s EC-Lab software.

### 3.3. Measurement and Data Analysis

For single tests, one sensor was used per test at a single current density. The open circuit potential (OCP), where no current passes in the system, was recorded for 30 s. The OCP ensures that the solution reaches an equilibrium state prior to introducing a constant current. After OCP, the potentiostat applies a constant current between the working and counter electrodes for 15 s through a chronopotentiometry step. A large range of current densities (60–960 A/m^2^) were tested. The current density was based on the area of the working electrode. For reusability tests, fresh 5 mL chloride solutions were tested with a reused sensor at room temperature. The reusability tests were run for 3 s, or until the 0.9 V cutoff was reached after a 30 s OCP.

For sweep tests, one sensor was used per test at multiple current densities. After an initial OCP step recorded for 60 s, a constant current density of 960 A/m^2^ was applied between the working and counter electrodes, followed by 240 A/m^2^, and lastly, 60 A/m^2^. Each chronopotentiometry step was run for 3 s, with a 60 s OCP step run between to let the system relax back to equilibrium. A potential cutoff was imposed on the system, such that if a potential greater than 0.9 V was reached, the system would move to the next step. For both single and sweep tests, EC Lab recorded the potential of the working electrode relative to the reference electrode (E_W_) as a function of time. A Python program determined the derivative of the potential with respect to time. To determine the transition time, the program found the maximum value of the potential derivative after an initial potential spike of 0.1 s. The corresponding time at this maximum was the transition time.

### 3.4. Electrode Surface Characterization

Sensors were submerged in 5 mL of chloride solutions to prevent the surface from being damaged from chip holder removal. The sensors were not washed prior to imaging, and may have had residual NaCl and Na_2_SO_4_ located on the surface. SEM images were captured with a Field Emission SEM (Hitachi SU8000 In-Line, Tokyo, Japan) in secondary electron detection mode. In addition to SEM, EDS (Bruker Quantax 200, Billerica, MA, USA) was used to elementally identify the surface of the working electrode. For both SEM and EDS, a 10 μA emission current and 10 kV acceleration voltage were used.

## 4. Results and Discussion

### 4.1. Detection of Chloride ion Concentration Using Chronopotentiometry

**Figure 2:**
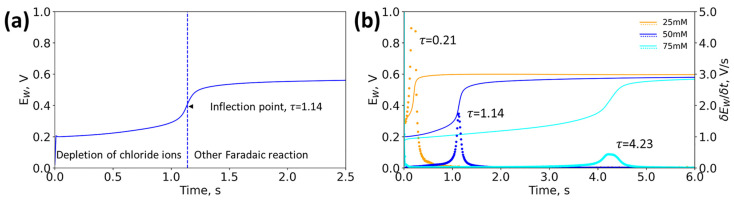
(**a**) Representative chronopotentiograms at 50 mM NaCl and 20 mM Na_2_SO_4_ at 25 °C in 240 A/m^2^. (**b**) The effect of concentration on the potential response and transition time at 25 °C in 240 A/m^2^. The dotted line represents the derivative of the chronopotentiogram.

We investigated chronopotentiometry to accurately detect chloride ion concentrations in aqueous solutions in near real-time. Figure 2a shows a representative chronopotentiogram of a 50 mM NaCl solution at a current density of 240 A/m^2^. Initially, the potential difference (E_W_) between the working and reference electrodes was close to zero. Upon applying a current density of 240 A/m^2^, the potential rose rapidly, which corresponded to the ohmic drop across the electrolyte [27]. Next, the chloride ions began to deplete near the surface of the working electrode, and this process slowed the voltage rise. At 1.14 s, the local chloride became completely depleted, and the potential rapidly rose, creating an inflection point in the chronopotentiogram as the system switched to another faradaic reaction. By taking the derivative of the chronopotentiogram, the time to reach the inflection point could easily be identified as the transition time, indicated by the dotted blue line in Figure 2a. The transition time provides a clear indication of chloride depletion, independent from an absolute potential value, and thus simple electrodes with a pseudo-reference can be employed.

The transition time is concentration dependent, and can be exploited as a sensor signal for quantifying urinary chloride. Figure 2b shows the relationship between chloride concentration and transition time at 240 A/m^2^. As the concentration of sodium chloride increased from 25 to 75 mM, the transition time increased from 0.21 to 4.23 s, and the derivative peak decreased in signal intensity. The increase in transition time was a result of the presence of more chloride ions at the working electrode surface, requiring a longer time to deplete. If the transition time exceeded 15 s, the signal was unable to be resolved. When the chloride concentration became too high (>125 mM) or low (<25 mM) at 240 A/m^2^, no inflection point was present in the chronopotentiogram, and the sensor could not detect the chloride concentration. The transition time does not occur because the slow rate of reaction was unable to fully deplete the chloride ions in samples of high chloride concentrations. Alternatively, at low chloride concentrations, the transition time occurred so quickly (e.g., <10 ms) that it was unresolvable by our instrumentation.

### 4.2. Current Density Optimization for Detection of Biologically Relevant Chloride Concentration

**Figure 3:**
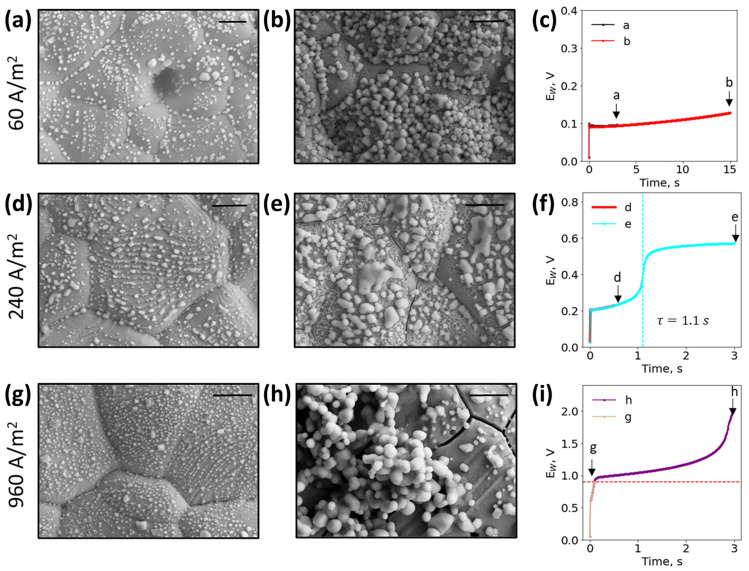
Scanning electron microscopy images of 50 mM NaCl and 20 mM Na_2_SO_4_ at 25 °C at (**a**) 60 A/m^2^ after running for 3 s and (**b**) after 15 s; at 240 A/m^2^ for (**d**) 0.5 s prior to transition time and (**e**) 3 s after transition time; at 960 A/m^2^ with a (**g**) voltage cutoff of 0.9 V and (**h**) without a voltage cutoff running for 3 s. Each experiment was used with a fresh screen-printed electrode. The corresponding chronopotentiogram for the 60 A/m^2^, 240 A/m^2^, and 960 A/m^2^ experiments are found in (**c**,**f**,**i**), respectively. The potential cutoff is represented by a horizontal red line. The transition time is represented by a vertical cyan line. All scale bars are 2.5 µm.

The proper selection of current density is critical to optimize sensor performance at a specific chloride concentration across the biologically relevant range of urinary chloride concentrations. Here, we optically and electrically characterized the working electrode surface at 60, 240, and 960 A/m^2^ across different time points in simple solutions of 50 mM NaCl, as shown in Figure 3. The EDS spectral data confirm the materials of a bare electrode and the presence of silver chloride in each SEM image, and can be found in Supplementary Figures S1–S2. It should be noted that the reference electrode was unaffected by the reactions occurring on the working electrode as shown in Supplementary Figure S3. When the chloride ion depletion occurred at the slow rate of 60 A/m^2^, as shown in Figure 3a–c, the faradaic reaction never fully depleted the local chloride ions, and no transition time at this chloride concentration was observed. The silver working electrode, shown in grey, continually had white silver chloride particles deposit on the surface as the reaction continued. At 240 A/m^2^, as shown in Figure 3d–f, the current density was optimal for this chloride concentration, and the chronopotentiogram resolved the transition time at 1.1 s. Prior to the transition time, the working electrode surface looked similar to Figure 3a. After the transition time, larger silver chloride particles were visible, and likely formed between 0.5–1.1 s. As the current density further increased to 960 A/m^2^, as shown in Figure 3g–i, local chloride ions quickly depleted, resulting in a rising potential, and the working electrode became damaged with cracks around the silver grain boundaries (Figure 3h). A potential cutoff that stopped the experiment when 0.9 V was reached prevented sensor damage, as shown in Figure 3g. By imposing the potential cutoff, unwanted reactions that affected the sensor’s working electrode surface were eliminated.

**Figure 4:**
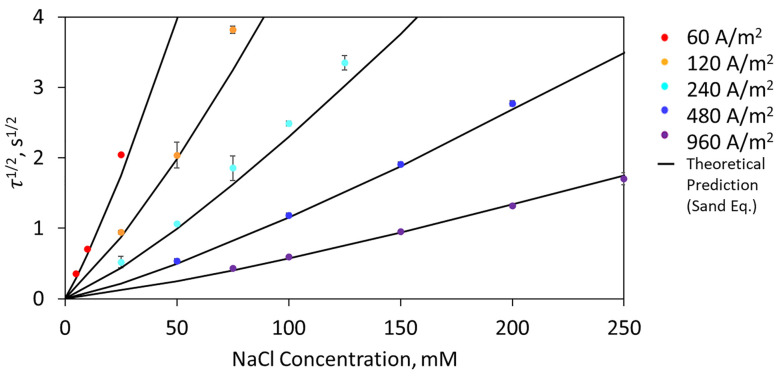
The experimental and theoretical (black line) square root of transition time (N = 3) as a function of chloride concentration in 20 mM Na_2_SO_4_ at 25 °C. One current density does not resolve the entire relevant concentration range of 5–250 mM NaCl.

By varying current density, we can resolve two orders of magnitude of chloride concentrations. If a particular chloride concentration could be resolved by a specific current density (60, 120, 240, 480, 960 A/m^2^), the resulting average square root of transition time τ^1/2^ was plotted as a function of NaCl concentration (5–250 mM), and is shown in Figure 4. Specific chloride concentrations were able to be resolved with different current densities. For example, 50 mM NaCl could be resolved at 120, 240, and 480 A/m^2^ at 2.03, 1.06, and 0.53 s^1/2^, respectively. By increasing the current density, the transition time could be decreased by increasing the chloride depletion reaction. Although a single current density was unable to resolve the entire range of biologically relevant chloride concentrations, we demonstrated optimal current densities for specific concentration ranges in near real-time (<15 s).

Furthermore, the Sand equation (Equation (2)) was able to accurately predict the sensor’s response. The theoretical transition time was based on a chloride ion diffusion coefficient of 1.6 × 10^−9^ m^2^/s, and the transference numbers were calculated using Equation 3 for each chloride ion concentration (5–250 mM NaCl) in the presence of 20 mM Na_2_SO_4_. The ion mobility (λ) used for Na^+^, Cl^−^, and SO_4_^2−^, were 5.01, 7.63, and 15.96 mS·m^2^/mol, respectively [41] (refer to Theory section). Based on the mobility and chloride ion concentration, the transference number was calculated to be between 0.05 and 0.49. The theoretical square root of transition time, calculated from Equation (2), predicted the experimental values, and is shown for each current density curve as a black line in Figure 4. For short transitions (τ^1/2^ < 2 s^1/2^), the agreement between theory and experiments was quite good. At longer transition times (τ^1/2^ > 3 s^1/2^), there was a greater deviation from the predicted values, resulting from the influence of convection. Additionally, slight deviations could be attributed to an incorrect assumption of the area of the working electrode (e.g., surface roughness), deviation in chloride ion concentration due to measurement error, or inaccuracies in calculated mass transport parameters, as they reflected an ideal case. With promising theoretical agreement, the entire range of biologically relevant chloride concentration can be resolved through the selection of optimal current densities.

### 4.3. Detection of Chloride ion Concentration Using Swept Current Densities

**Figure 5:**
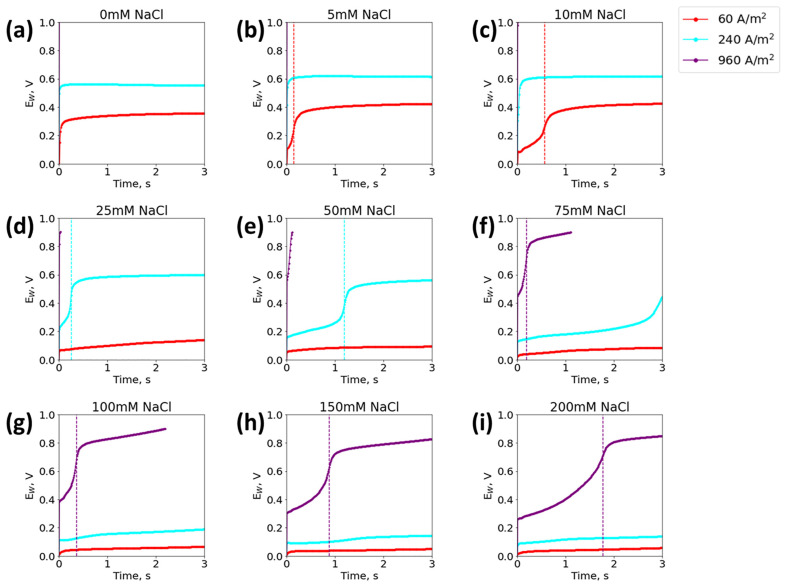
Chronopotentiogram of each mode of sweep test run in succession at 3 s for 960 A/m^2^,480 A/m^2^, and 60 A/m^2^ in 20 mM Na_2_SO_4_ at 25 °C in NaCl concentrations of (**a**) 0 mM, (**b**) 5 mM, (**c**) 10 mM, (**d**) 25 mM, (**e**) 50 mM, (**f**) 75 mM, (**g**) 100 mM, (**h**) 150 mM, and (**i**) 200 mM. Vertical dotted lines in each plot indicate the transition time. Prior to each constant current density step, the system was run at OCP for 1 min.

To improve the detection range of a single sensor, a sweep algorithm was developed using multiple current densities on a single sensor, but limiting the potential to a predetermined potential cutoff value. The resulting chronopotentiograms of different chloride concentrations sensed with the sweep method are plotted in Figure 5. Each sweep cycle corresponds to a different resolvable chloride concentration range; the high (75–200 mM), mid (15–75 mM) and low ranges (5–15 mM) were resolved with 960, 240, and 60 A/m^2^, respectively (note that the 250 mM NaCl concentration could be resolved if the experiment time was increased). To sweep current density while still maintaining the integrity of the sensor, the sensor had to be swept from high to low current densities. In this configuration, a high chloride concentration (<75 mM) either resolved immediately, or a low concentration quickly hit the potential cutoff, as seen in the purple curve of Figure 5a–e. If swept from low to high current density, the sensor quickly became covered with silver chloride in the presence of a high chloride concentration, and the measurement became unreliable (as demonstrated from Figure 3a–c). Ideally, the sensor would stop sweeping as soon as the transition time was detected, in order to prevent further change to the surface of the working electrode. The swept τ^1/2^ were in good agreement (2.6–7.3% difference) with the experimental τ^1/2^ found by the single current density method, and comparison tables can be found in Supplementary Tables S1 and S2. The proposed sweep method resolved the large range of relevant urinary chloride concentrations with a single sensor.

**Figure 6:**
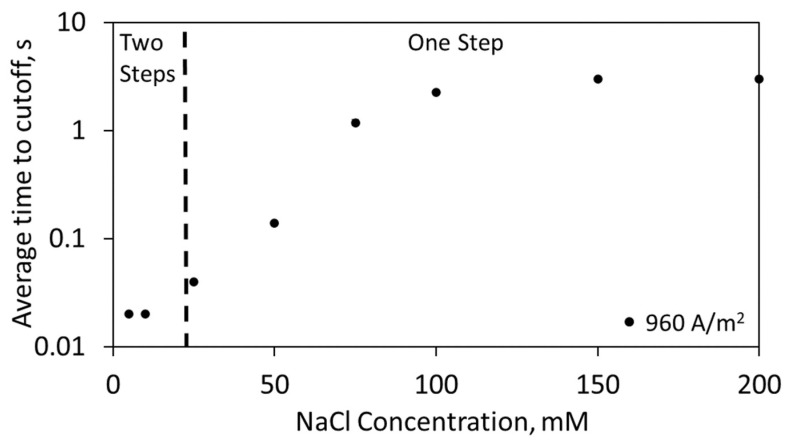
Average time to potential cutoff when run for 3 s at 960 A/m^2^ in 20 mM Na_2_SO_4_ at 25 °C. Note that 150 and 200 mM NaCl never reached the cutoff. The sweep methodology only required 1 step to resolve 25 to 200 mM NaCl. Note that the time to potential cutoff was used as the signal for 25–75 mM, and the transition time was used to resolve 75–200 mM. For 5 to 15 mM, only two sweep steps were needed.

Additional sensor response information can be used to improve the sweep algorithm for faster chloride quantification. As shown in Figure 6, the time to reach the potential cutoff is an important metric that can help determine how to enact the sweep algorithm. By incorporating the time to potential cutoff metric, 25 mM to 250 mM was identified using a single sweep step of 960 A/m^2^. The signal was either determined with the time to potential cutoff (25–75 mM) or the transition time (75–250 mM). In cases where there were low chloride concentrations (<15 mM), the time to reach the potential cutoff was approximately 0.02 s to 0.03 s, and the sensor was unable to distinguish between these different concentrations. In this case, the sensor would have two sweep steps (960 A/m^2^, 60 A/m^2^), thus resolving the low concentrations and reducing the total experimental sensing time from the present three-sweep configuration. Additionally, the potential of the sensor at early times within the experiment (e.g., 0.2 s) could be used as an indicator of chloride concentration, as the potential is a function of chloride concentration (Supplementary Figure S5). However, absolute potential as an indicator should be treated with caution, as many parameters (e.g., conductivity) affect the potential of the sensor and could lead to inaccurate and unstable readings, as the sensor contains a pseudo-reference electrode. Using additional metrics, the sweep algorithm can be shortened to resolve the large range of relevant chloride concentrations in near real-time (3–66 s).

### 4.4. Sensor Reusability

**Figure 7:**
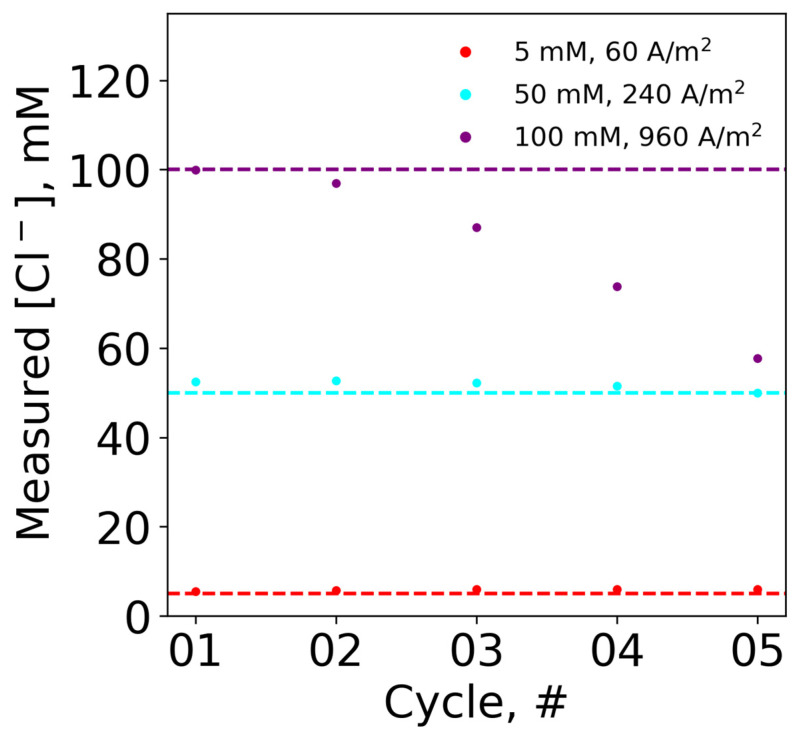
The measured chloride concentration, from back calculation using transition time, versus cycle number, using fresh 5 mL solutions with a reused sensor with 5 mM NaCl at 60 A/m^2^, 50 mM NaCl at 240 A/m^2^, and 100 mM at 960 A/m^2^, in solutions of 20 mM Na_2_SO_4_ at room temperature, run for 3 s, or until the 0.9 V cutoff was reached.

To extend the lifetime of a single sensor, reusability was explored for cases where multiple measurements are needed. The same electrode in fresh sodium chloride solutions was tested for five experimental cycles. The experiments were conducted at different chloride concentrations at the optimal current density for each concentration (5 mM at 60 A/m^2^, 50 mM at 240 A/m^2^, and 100 mM at 960 A/m^2^), and with the potential cutoff. Based on the resulting experimental transition times, the measured chloride concentrations were back calculated using Equations (2) and (3) and compared to the actual chloride concentrations (i.e., horizontal dashed line). The measured chloride concentration was plotted as a function of experimental cycles, as shown in Figure 7. For both 5 and 50 mM NaCl, the sensor was able to be reused across the five experimental cycles, with minimal deviation from the actual chloride concentrations. However, at the higher concentration of 100 mM NaCl, the sensor was only able to be used twice before significantly drifting from the actual chloride concentration values. Specifically, the measured chloride values at the first and third cycles were 99.9 mM and 87 mM, respectively. Thus, the chloride concentration at optimal current density affected the reusability of the sensor.

**Figure 8:**
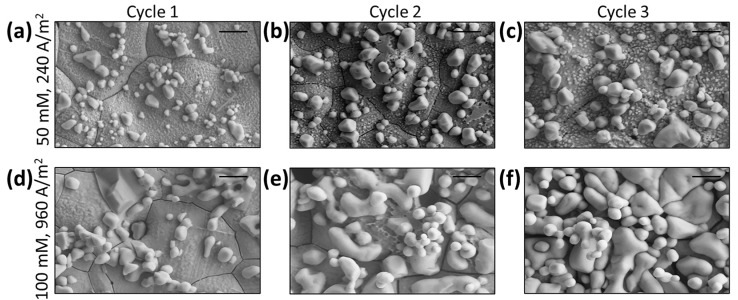
SEM images of sensors after running for (**a**,**d**) 1 cycle; (**b**,**e**) 2 cycles; and (**c**,**f**) 3 cycles, with experimental conditions of 50 mM NaCl, 240 A/m^2^ and 100 mM NaCl, 960 A/m^2^, respectively. Each cycle used 5 mL of fresh 20 mM Na_2_SO_4_ simple solution at 25 °C. A new electrode was used for each test, and was stopped after different cycle numbers. All scale bars are 2.5 µm.

The surface of the working electrode was further characterized using SEM and EDS to explore the impact of both chloride concentration and the current density on the reusability of the sensor. As shown in Figure 8, the SEM images depict the surface of the working electrode in both 50 mM and 100 mM NaCl solutions at the end of three consecutive chronopotentiometry cycles. The EDS results support the hypothesized formation of silver chloride, which can be found for each SEM image in Supplementary Figure S4. In both cases, more silver chloride was formed on the working electrode surface after each cycle. At 50 mM, the surface of the working electrode was still exposed to the fresh sodium chloride solution after three cycles. In contrast, the 100 mM NaCl solutions had a working electrode surface that was covered with silver chloride after two cycles. The 100 mM NaCl solution at 960 A/m^2^ had a different silver chloride morphology, with particles of larger surface area than the solution with 50 mM NaCl at 240 A/m^2^. The silver chloride was likely blocking the active silver, or changing the surface properties of the electrode such that the transition time deviated from the predictions of the Sand equation (Equation (2)). Thus, the sensor was reusable in cases of low chloride concentration (≤50 mM), where the silver chloride particle morphology had a smaller surface area. For future miniaturization of the sensor, many chloride sensors could be used in series to monitor a patient’s inter/intraday urinary chloride concentration.

### 4.5. Effect of Relevant Clinical Parameters on Transition Time

**Figure 9:**
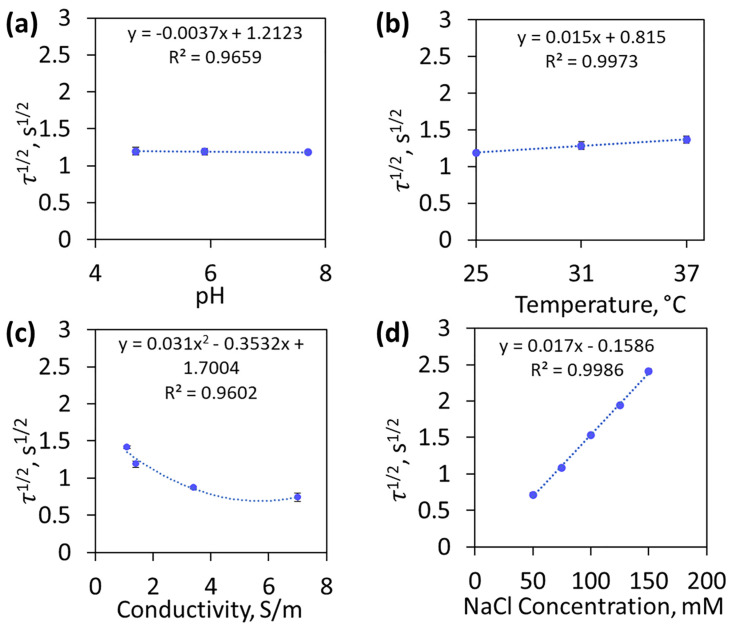
The effect of (**a**) pH at 20 mM Na_2_SO_4_ and 100 mM NaCl at 25 °C; (**b**) temperature in 20 mM Na_2_SO_4_ and 100 mM NaCl; and (**c**) conductivity with varying Na_2_SO_4_ concentrations and 100 mM NaCl at 25 °C with respect to average squared root of transition time (N = 3) in 480 A/m^2^. The effect of other ions was investigated with (**d**) NaCl-spiked artificial urine as a function of the average square root of transition time (N = 3) at 480 A/m^2^ and 25 °C. Each curve was fit with a regression line (blue dotted line).

The chloride sensor was evaluated in more complex solutions, and was found to be relatively independent of pH and temperature. These solution parameters were evaluated in simple solutions of 100 mM NaCl at 480 A/m^2^, and the results can be found in Figure 9a,b. To illustrate the impact of pH and temperature, the plot was fitted with a linear regression trendline, as shown in Figure 9a,b. It should be noted that 100 mM NaCl was chosen as a representative of normal urinary chloride, and could be quickly resolved with the current density of 480 A/m^2^. The relevant pH range of urine is 4 to 8, and the experimental τ^1/2^ did not significantly change as a function of pH, as hydrogen concentration does not influence the depletion of chloride ions near the working electrode. The solution temperature was considered for different temperatures (25, 31, 37 °C), and had minimal effect on τ^1/2^. Temperature affected the diffusion coefficient, D, which increased 2–3% per degree [27]. The theoretical and average experimental τ^1/2^ had a 1.9–3.1% difference when the diffusion coefficient was adjusted by 3% per degree, which was a significant improvement over the 3.1–17.2% difference with no adjustment. To adjust for the changing diffusion coefficient, readily available temperature sensors [44] could be incorporated into the future design of the urinary chloride sensor.

Conductivity and transference number had a greater impact on the sensor transition time, and should be considered in future chloride sensor design. In Figure 9c, the conductivity was adjusted by varying the Na_2_SO_4_ concentration (5–500 mM), and ranged from 1.1 –7 S/m. In these experiments, conductivity was indirectly proportional to the transference number (0.52–0.04), and the data were fitted with a second-order polynomial regression trendline. Higher conductivities increased the fraction of total ions in solution that were not chloride, thus decreasing the chloride ion transference number. At 500 mM Na_2_SO_4_ and 7 S/m, the transference number was negligible, and the percent difference between the experimental and theoretical τ^1/2^ was 0.2%. When the conductivity was decreased (1.1–3.4 S/m), there was a slightly higher percent difference (3–8%). To account for the effect of varying the transference number, conductivity sensors [45] could be incorporated into the design to enable calculation of transference number variations. Additionally, more non-chloride-containing salt solutions (e.g., Na_2_SO_4_) could be added to the urine sample prior to testing. Adding additional ions will increase the solution conductivity and decrease the fraction of chloride present in the total solution, making the transference number negligible. The elimination of the uncontrollable transference number in the Sand equation (Equation (2)) would simplify the sensor algorithm in complex solutions where the ions change over time (e.g., urine).

The chloride sensor was operated in the presence of other ions relevant to applications in urinary chloride. Sodium iodide was shown to have no effect on the sensor signal (Supplementary Figure S6). The effect of other ions was evaluated by varying chloride concentrations (50–200 mM) in purchased artificial urine solutions, as shown in Figure 9d. The sensor was able to resolve 50 to 150 mM NaCl, but could not resolve 200 mM after 15 s of applied current. This deficiency was attributed to the conductivity difference of the simple and artificial solutions with 1.4 S/m and 1.2 S/m, respectively, in 100 mM NaCl. The lower conductivity of the artificial urine sample increased the transition time, and agrees with the trend described in Figure 9c. The presence of other ions in the artificial urine did not affect the sensor’s ability to resolve the transition time.

## 5. Conclusions

The chloride sensor described in this research uses chronopotentiometry, combined with simple commercially available screen-printed electrodes, to resolve the entire range of relevant urinary chloride concentrations in near real-time, with no chemical modification or added reagents. The transition time serves as a reliable and clear indicator of bulk chloride concentration, and can be easily tuned by changing the applied current density. By optimizing the applied current density, the relevant chloride concentration range was resolved using a single sensor with a current density sweep algorithm, and the sensor lifetime was extended through reuse in cases of low chloride concentration (≤50 mM). The pH and temperature were shown to have a minimal impact on the sensor’s response, with the conductivity and transference number having a greater influence. Chloride-spiked artificial urine was successfully quantified, and demonstrates the sensor’s feasibility for clinical applications. We believe this research can trigger additional research to further develop the sensor by investigating the effects of interfering species (e.g., halides, organics), and evaluating the feasibility in real urine samples. Sensor performance can be further improved by optimizing the sweep algorithm to further reduce detection time and account for sensor lifetime; furthermore, by incorporating additional sensors (i.e., conductivity, temperature) shifts in the transition time can be corrected. The sensor also has the capability of being integrated easily into an existing uroflowmeter to provide actionable data for critically ill patients in the ICU. The resulting simple, portable, user-friendly sensor provides a feasible path for achieving near real-time urinary chloride quantification in a variety of clinical settings.

## 6. Patents

The work described herein is the subject of an invention report submitted to the University of Michigan’s technology transfer office and is currently patent pending.

## Data Availability

The data that support the findings of this study are available from the corresponding author upon reasonable request.

## Supplementary Materials

The following supporting information can be downloaded at: https://www.mdpi.com/article/10.3390/bios13030331/s1, Figures S1–S4: Elemental analysis of unused and tested electrodes; Tables S1 and S2: Transition times and measured chloride in sweep experiments; Figure S5: Potential values in sweep experiments; Figure S6: Iodide interference [46].
