# Supplementary material for: Electrochemical Sensing of Urinary Chloride Ion Concentration for Near Real-Time Monitoring
Source: Biosensors (Basel). 2023 Feb 28;13(3):331. doi: 10.3390/bios13030331 (PMC10046868; doi:10.3390/bios13030331)
Supplement: Supplementary file 1 [file biosensors-13-00331-s001.zip › biosensors-2190626-supplementary.pdf]

biosensors

MDPI

Supplementary Material

Electrochemical Sensing of Urinary Chloride Ion Concentration for Near Real-Time Monitoring

Anna M. Nelson [1], Sanaz Habibi [1], John O. L. DeLancey [2], James A. Ashton-Miller [3,4] and Mark A. Burns [1,4,*]

[1] Department of Chemical Engineering, University of Michigan, Ann Arbor, MI 48109, USA
[2] Department of Obstetrics & Gynecology, University of Michigan, Ann Arbor, MI 48109,USA
[3] Department of Mechanical Engineering, University of Michigan, Ann Arbor, MI 48109, USA
[4] Department of Biomedical Engineering, University of Michigan, Ann Arbor, MI 48109, USA
* Correspondence: maburns@umich.edu

S.1 Elemental Analysis of Unused and Tested Electrodes

The elemental analysis of the screen printed electrodes was conducted by using scanning electron microscopy (SEM) and Energy Dispersive X-ray Spectroscopy (EDS) with a Bruker Quantax 200. As a baseline, an unused electrode was analyzed via SEM-EDS as shown in Figure S1. The SEM-EDS confirmed the working and reference electrodes to be silver, the counter electrode to be carbon and the barrier layer to be an aluminum oxide. The EDS information for the corresponding surface characterization present in Figure 3 of the manuscript can be found in Figure S2. In each spectra, a strong presence of Ag and Cl indicate the formation of AgCl. In addition, other trace elements of aluminum, sodium, oxygen and sulfur can be observed. These are likely from the barrier layer and dried chemicals (e.g., NaCl, Na_2SO_4) that precipitated onto the sensor surface when removed from the test solution. Additionally, as shown in Figure S3, the reference electrode was unaffected by the reaction on the working electrode. In reusability experiments, the corresponding EDS to Figure 8 in the manuscript can be found in Figure S4. The EDS indicates an increase in AgCl formation in both 50 mM and 100 mM NaCl as shown by the increase in normalized weight percent of chlorine from 9.47% to 15.37 % and 0.23% to 14.98%, respectively.

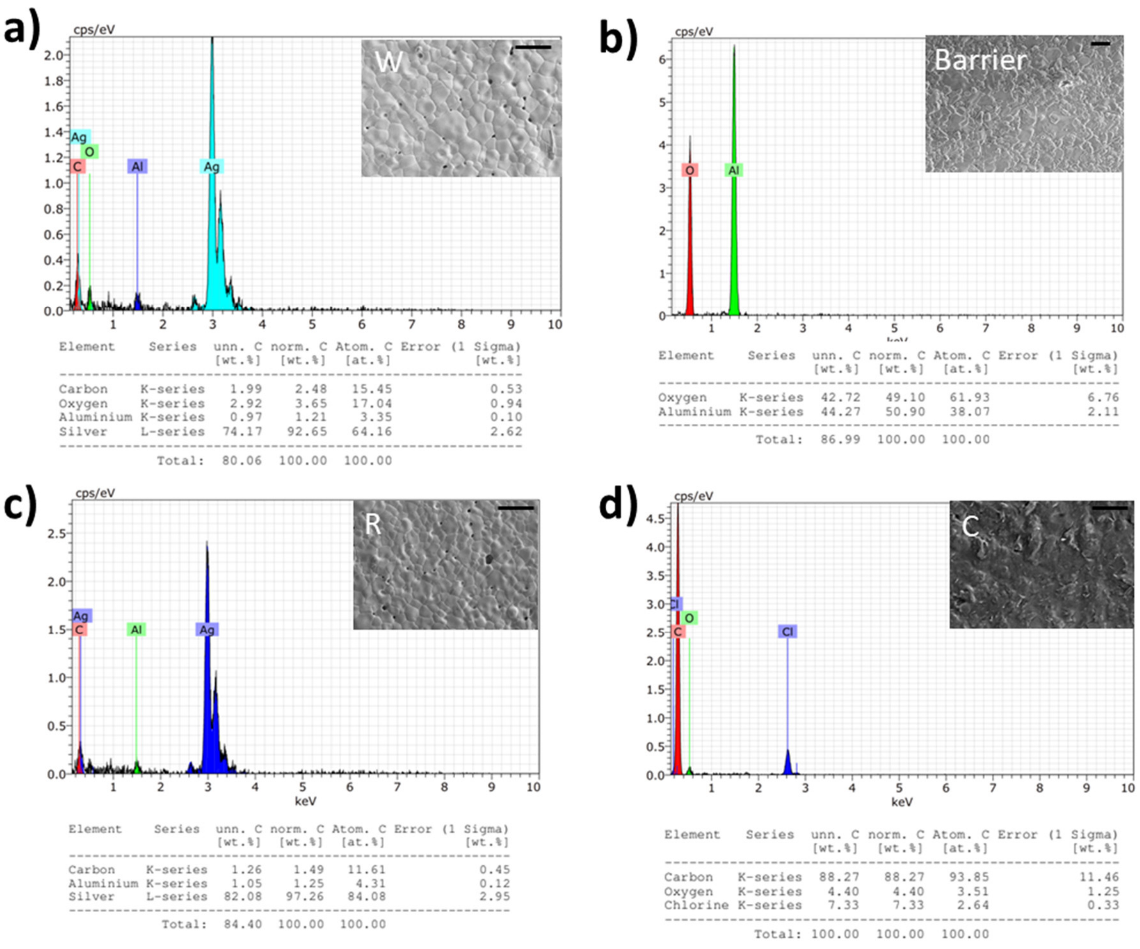

Figure S1. SEM/EDS of **a)** the working electrode **b)** the barrier film **c)** the reference electrode and **d)** the counter electrode of a bare Dropsens C013 electrode. All scale bars are 25 μm.

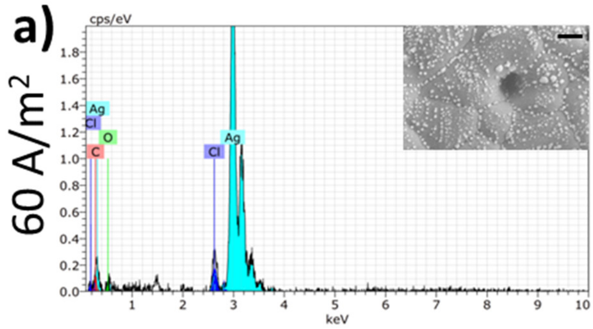

Element	Series	unn. C [wt.%]	norm. C [wt.%]	Atom. C [at.%]	Error (1 Sigma) [wt.%]
Carbon	K-series	0.78	0.88	6.58	0.30
Oxygen	K-series	1.35	1.51	8.50	0.59
Chlorine	K-series	1.85	2.08	5.28	0.13
Silver	L-series	85.05	95.53	79.65	2.99
	Total:	89.03	100.00	100.00	

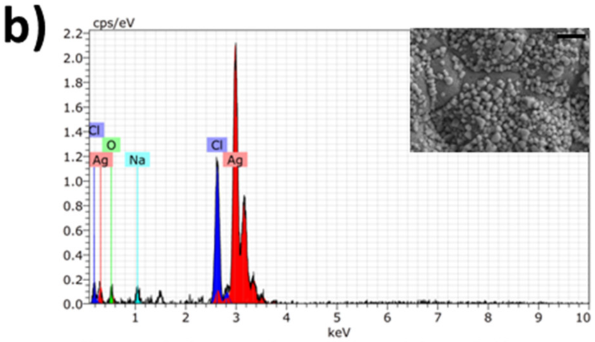

Element	Series	unn. C [wt.%]	norm. C [wt.%]	Atom. C [at.%]	Error (1 Sigma) [wt.%]
Silver	L-series	72.20	82.91	57.01	2.54
Oxygen	K-series	1.97	2.27	10.50	0.70
Chlorine	K-series	11.78	13.53	28.30	0.48
Sodium	K-series	1.13	1.30	4.19	0.13
	Total:	87.08	100.00	100.00	

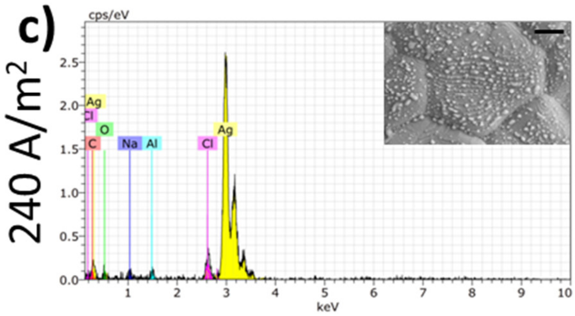

Element	Series	unn. C [wt.%]	norm. C [wt.%]	Atom. C [at.%]	Error (1 Sigma) [wt.%]
Carbon	K-series	0.66	0.75	5.22	0.30
Oxygen	K-series	1.88	2.12	11.13	0.78
Sodium	K-series	0.86	0.97	3.55	0.13
Aluminium	K-series	0.70	0.79	2.45	0.09
Chlorine	K-series	1.89	2.14	5.06	0.14
Silver	L-series	82.47	93.23	72.58	2.93
	Total:	88.45	100.00	100.00	

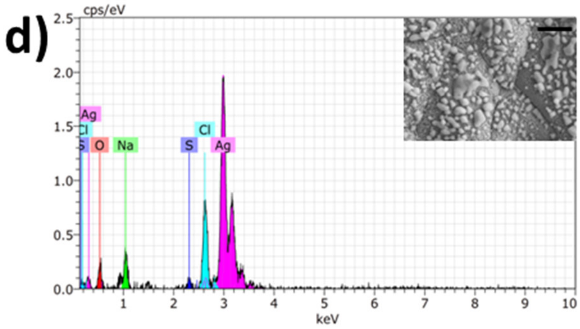

Element	Series	unn. C [wt.%]	norm. C [wt.%]	Atom. C [at.%]	Error (1 Sigma) [wt.%]
Oxygen	K-series	4.44	5.08	20.71	1.31
Sodium	K-series	3.54	4.05	11.51	0.31
Sulfur	K-series	0.78	0.89	1.82	0.09
Chlorine	K-series	8.13	9.30	17.13	0.38
Silver	L-series	70.52	80.67	48.83	2.53
	Total:	87.41	100.00	100.00	

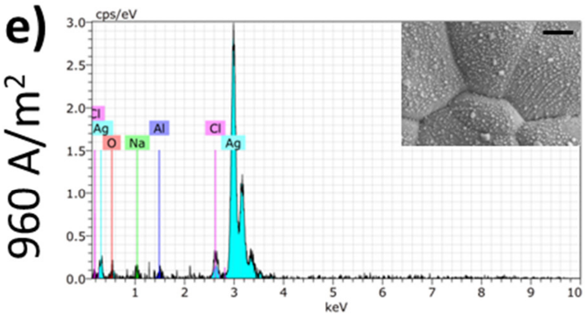

Element	Series	unn. C [wt.%]	norm. C [wt.%]	Atom. C [at.%]	Error (1 Sigma) [wt.%]
Oxygen	K-series	1.63	1.78	10.04	0.77
Sodium	K-series	0.95	1.04	4.09	0.14
Aluminium	K-series	0.65	0.71	2.39	0.09
Silver	L-series	86.84	94.84	79.36	3.11
Chlorine	K-series	1.48	1.62	4.12	0.13
	Total:	91.56	100.00	100.00	

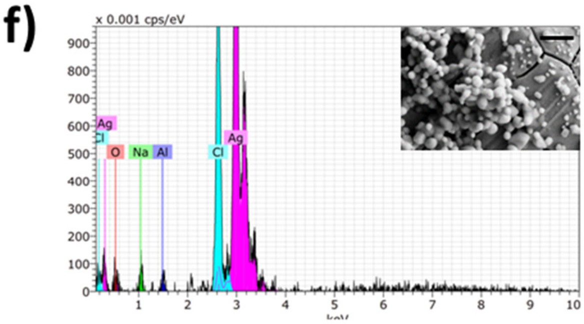

Element	Series	unn. C [wt.%]	norm. C [wt.%]	Atom. C [at.%]	Error (1 Sigma) [wt.%]
Oxygen	K-series	1.24	1.45	6.66	0.63
Sodium	K-series	0.88	1.03	3.29	0.13
Aluminium	K-series	0.31	0.36	0.98	0.07
Chlorine	K-series	14.23	16.60	34.33	0.60
Silver	L-series	69.02	80.55	54.74	2.50
	Total:	85.69	100.00	100.00	

Figure S2. Corresponding EDS to SEM images shown in Figure 3 of the Manuscript of 50 mM NaCl and 20 mM Na_2SO_4 at 25 °C at **a)** 60 A/m² after running for 1s and **b)** after 15s. At 240 A/m² for **c)** 0.5s and **d)** 3s. At 960 A/m² with a **e)** voltage cutoff of 0.9V and **f)** without a voltage cutoff running for 3 s. All scale bars 2.5 μm.

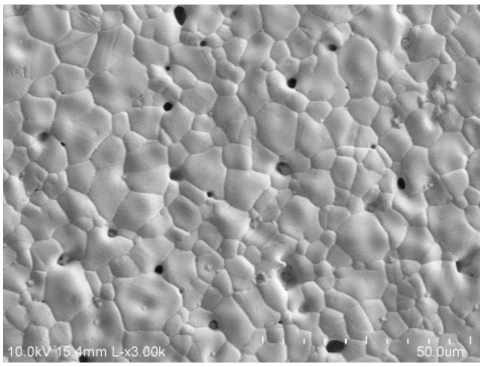

Figure S3. SEM image of the reference electrode after a solution of 50 mM NaCl and 20 mM Na_2SO_4 was tested at 25 °C with 960 A/m^2.

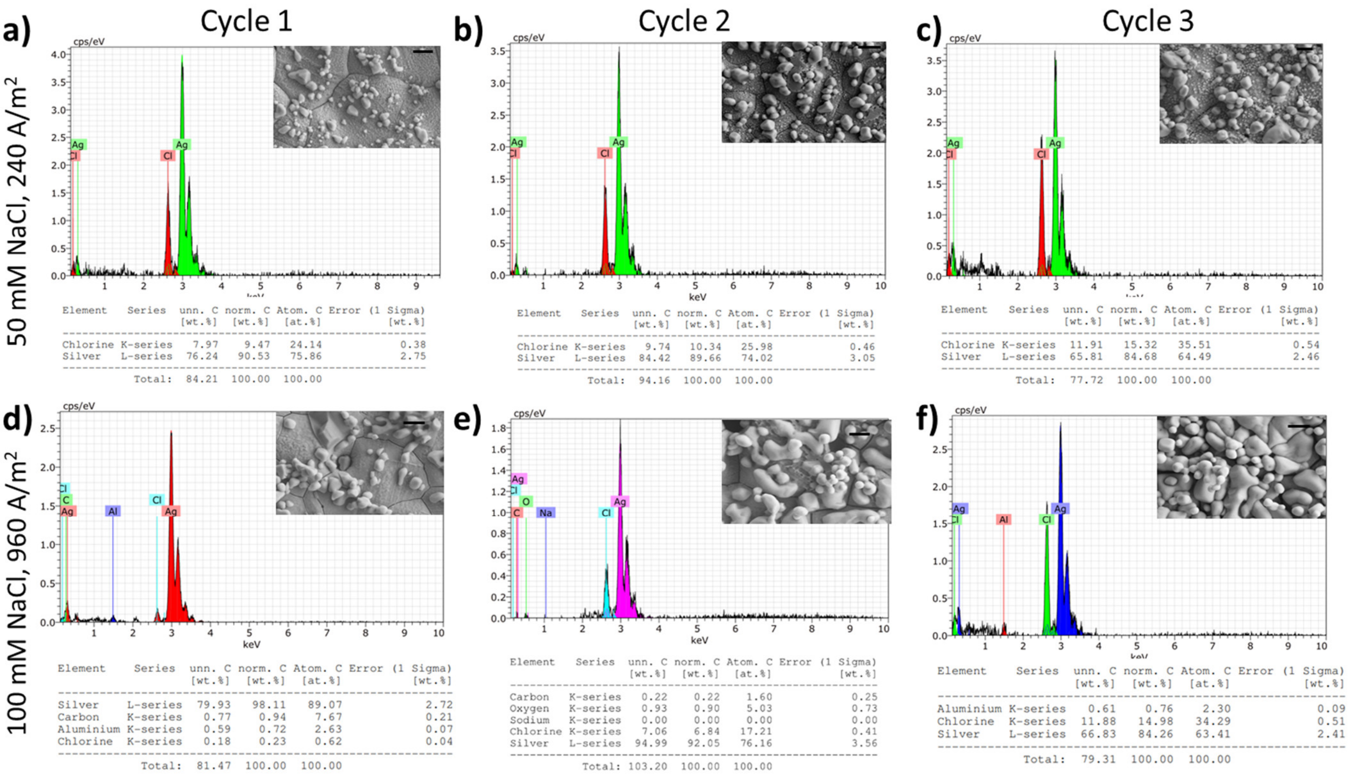

Figure S4. EDS corresponding to manuscript Figure 8 of reused sensors in 50 mM NaCl and 240 A/m^2 after running for **a)** and **d)** 1 cycle, **b)** and **e)** 2 cycles and **c)** and **f)** 3 cycles in 50 mM NaCl, 240 A/m^2 and 100 mM NaCl, 960 A/m^2 respectively. Each cycle used 5 mL of fresh 20 mM Na_2SO_4 simple solution at 25℃. All scale bars are 2.5 µm.

S.2 Transition Time and Measured Chloride in Sweep Experiments

To compare single electrode and sweep experiments, the average square root of transition time was compiled for each technique in Table S1. The average measured chloride concentration was then back calculated using the Sand Eq. and the values for both methods can be found in Table S2. The values of both the single and sweep methods are comparable with minimal percent difference (≤ 7.3%).

Table S1. Comparison of the square root of transition time of tests conducted with an individual electrode versus tests completed with the sweep method in various NaCl concentrations (N=3) in 20 mM Na_2SO_4 at 25°C.

		Individual Electrode, $\tau^{\frac{1}{2}}$, $s^{\frac{1}{2}}$			Sweeped Electrode, $\tau^{\frac{1}{2}}$, $s^{\frac{1}{2}}$			
NaCl Concentration, mM	Conductivity S/m	960 A/m^2	240 A/m^2	60 A/m^2	960 A/m^2	240 A/m^2	60 A/m^2	% Difference
5	0.4	-	-	0.36	-	-	0.38	-7.3
10	0.5	-	-	0.70	-	-	0.73	-4.5
25	0.6	-	0.52	2.04	-	0.50	-	4.9
50	0.92	-	1.06	-	-	1.09	-	-2.6
75	1.1	0.44	1.85	-	0.44	-	-	0
100	1.4	0.60	2.49	-	0.60	-	-	0
150	2.0	0.95	-	-	0.95	-	-	0
200	2.5	1.32	-	-	1.32	-	-	0

Table S2. Comparison of the average measured chloride concentration, through back calculation in Sand equation, of tests conducted with an individual electrode versus tests completed with the sweep method in various NaCl concentrations (N=3) in 20 mM Na_2SO_4 at 25°C.

		Individual Electrode, $mM\ NaCl$			Sweeped Electrode, $mM\ NaCl$			
NaCl Concentration, mM	Conductivity S/m	960 A/m^2	240 A/m^2	60 A/m^2	960 A/m^2	240 A/m^2	60 A/m^2	% Difference
5	0.4	-	-	5.93	-	-	6.38	-7.3
10	0.5	-	-	11.21	-	-	11.73	-4.5
25	0.6	-	30.01	29.35	-	28.58	-	4.9
50	0.92	-	53.63	-	-	55.04	-	-2.6
75	1.1	80.74	85.80	-	80.74	-	-	0
100	1.4	103.68	2.49	-	103.68	-	-	0
150	2.0	152.33	-	-	152.33	-	-	0
200	2.5	195.86	-	-	195.86	-	-	0

S.3 Potential Values in Sweep Experiments

Potential can be used as an indicator of chloride concentration at 0.2 s of running the sweep experiments as shown in Figure S5. However, caution should be taken when using potential as a parameter to indicate chloride concentration as many factors can affect the potential value and a pseudo reference electrode is used in the system.

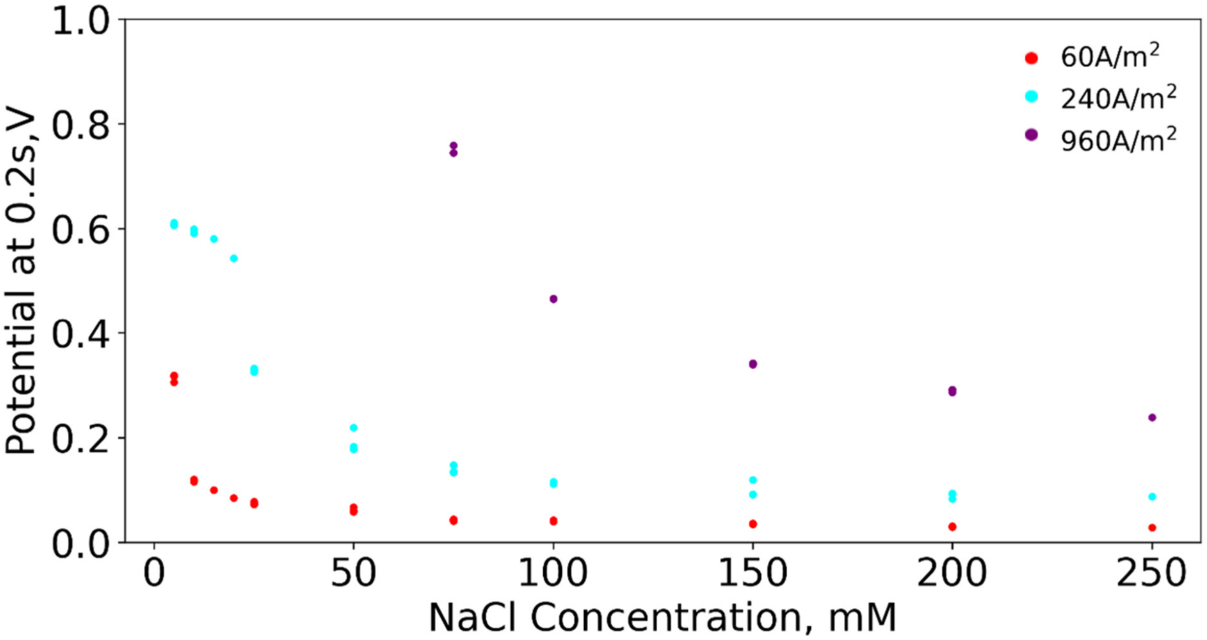

Figure S5. The potential value at 0.2 s during the sweep experiments for each current density step.

S.4 Iodide Interference

To investigate halide interference, we tested solutions that contained the clinically relevant concentration of sodium iodide [1]. In Figure S6, the chronopotentiometric signal of solution containing sodium iodide was compared to samples containing no sodium iodide. As shown, sodium iodide has no impact on the transition time.

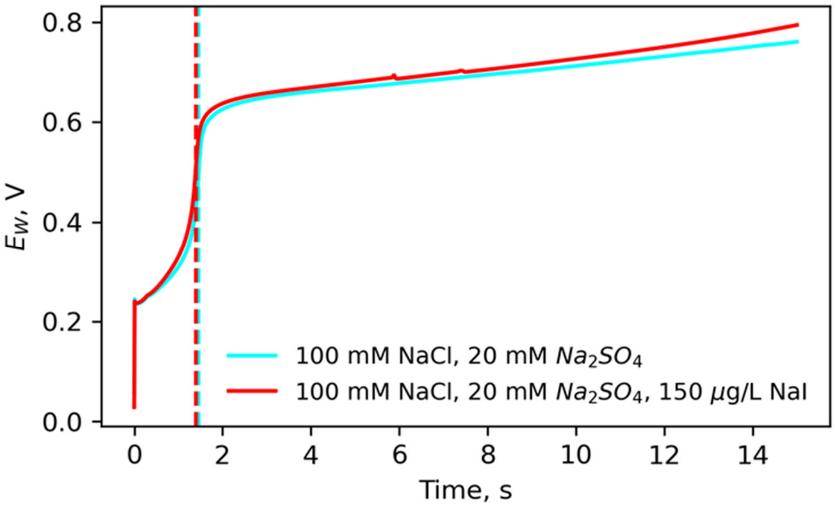

Figure S6. Chronopotentiogram of 100 mM NaCl, 20 mM Na_2SO_4 was tested at 25 °C with 480 A/m^2 with and without 150 µg/L NaI. The sodium iodide did not affect the signal. The dashed line represents the transition time for each curve.

Reference

1. Delange, F.; Benoist, B.d.; Bürgi, H. Determining median urinary iodine concentration that indicates adequate iodine intake at population level. *Bull. World Health Organ.* **2002**, *80*, 633–636.
